# Feminist approach to geriatric care: comprehensive geriatric assessment, diversity and intersectionality

**DOI:** 10.1007/s11019-021-10052-1

**Published:** 2021-09-16

**Authors:** Merle Weßel

**Affiliations:** Ethics in Medicine, Carl Von Ossietzky University of Oldenburg, Ammerländer Heerstraße 114-118, 26111 Oldenburg, Germany

**Keywords:** Geriatric medicine, Diversity, Intersectionality, Aging

## Abstract

Despite being a collection of holistic assessment tools, the comprehensive geriatric assessment primarily focuses on the social category of age during the assessment and disregards for example gender. This article critically reviews the standardized testing process of the comprehensive geriatric assessment in regard to diversity-sensitivity. I show that the focus on age as social category during the assessment process might potentially hinder positive outcomes for people with diverse backgrounds of older patients in relation to other social categories, such as race, gender or socio-economic background and their influence on the health of the patient as well as the assessment and its outcomes. I suggest that the feminist perspective of intersectionality with its multicategorical approach can enhance the diversity-sensitivity of the comprehensive geriatric assessment, and thus improve the treatment of older patients and their quality of life. By suggesting an intersectional-based approach, this article contributes to debates about justice and diversity in medical philosophy and advocates for the normative value of diversity in geriatric medicine.

## Introduction

In most Western societies, demographic changes are leading to an increased amount of older people. Low birth rates and higher life expectancies are changing the societal age ratio, resulting in a higher number and proportion of older people living longer. This is not without its challenges, as old age is often related to an increase of health issues and bodily degeneration. However, illness in old age is a complex phenomenon because the health issues older patients are facing often are not due to one specific cause but rather attributed to multiple causes. These include a decline of bodily functions, disease or illness, or social factors such as an unsuitable environment for their needs, which can lead to a decline in health and quality of life. Thus, the aging of the population poses ethical questions of adequate healthcare for older people (e.g., Ehni et al. 2018; Wareham 2018). Furthermore, demographic changes not only mean more older people but also a greater visibility of diversity in older people in terms of lifestyles and needs. This needs to be recognized in healthcare provided for older people to avoid inequality and increase the quality of life for older, diverse people (Weßel and Schweda 2021). However, this poses the question to what extent diversity should be taken into account in philosophical debates about healthcare for older people? Or if it is, as some scholars have argued, serves only as a descriptive tool and renders the attempt fruitless from a normative perspective (Bracanovic 2013; Ten Have and Gordijn 2011).

The traditional disease-orientated model used in medicine, focusing on the diagnosis and cure of a disease or aliment, does not fit the needs of older patients with their non-disease specific problems, such as multimorbidity, frailty, polypharmacy, and disability (Palmer and Onder 2018). Geriatrics is the medical discipline that focuses on these older patients and their health issues (Palmer and Onder 2018). The *comprehensive geriatric assessment* (CGA) is the central method to analyse the older patient as a whole, as it attempts to identify and quantify the needs of the patient at the physical, psychological, and social levels. The CGA then provides the basis for the development and implementation of a treatment plan. Although CGA aims at the multidimensional approach of care for older patients, too often it focuses only on the physical assessment and neglects the social aspects that affects the health of the patient. This is compounded by the standardized nature of the assessment’s tests which cannot fully comprehend older people’s subjective positions or their diversity. The result is that this potentially negatively hinders the outcome of the tests and affect the treatment plan of older patients. In this way, while CGA surely stands for a holistic approach to health and disease in old age, I suggest that it is ultimately not sensitive enough to tackle the diverse needs of older patients.

This paper critical examines the three steps of CGA—the diagnosis, the treatment plan and the implementation—utilising the feminist theory of intersectionality as a method to strengthen the assessment of diverse older patients. In this way, it provides a contribution to the feminist philosophy of geriatric medicine that focuses on the just treatment of older people in intersectional-inspired healthcare. Additionally, it draws attention to the social categories intersecting with age, such as gender, sexuality or class, which can lead to invisible discrimination of diverse older people in healthcare. Intersectionality was introduced by feminist theorists in the early 1990s to demonstrate that structural discrimination is not only a one-dimensional issue like racism or sexism, but might occur at intersections of different social categories, such as gender, race, and class (Crenshaw 1989, 1990). Research, especially studies on public health, have found intersectionality to be fruitful in identifying multidimensional, structural discrimination in the context of health and medicine (Hankivsky 2012; Hankivsky and Christofferson 2008; Bowleg 2012; Bauer and Scheim 2019).

This article contributes to the debates about aging people in medical philosophy primarily in two ways. Firstly, it draws attention to the problem of a homogenized understanding of older people. It suggests that this homogenization can lead to injustice and discrimination, because the needs of diverse individuals are not adequately addressed in healthcare. Secondly, I show the analytical importance of feminist intersectional views to understand the normative value of diversity in medical philosophy contributing to debates about justice and equality in healthcare. The paper contributes to discussions on diversity of aging in geriatric medicine and medical philosophy and suggests that the feminist theory of intersectionality and its awareness of structural discrimination and diversity might be a useful tool to strengthen normative perspectives on older people in medicine (Weßel and Schweda 2021).

## The intersectional complexity of illness in old age and comprehensive geriatric assessment

The term “geriatrics” was coined in 1909, but geriatric medicine was not institutionalized until the 1940s, after Majorie Warren found that the existing methods of care for older patients were not sufficient and demanded the development of a special geriatric unit (Warren 1948; Forciea 2008). Warren established an assessment model to evaluate the best care options for older patients, such as being in a nursing home, at a hospital, or a psychiatric facility (Warren 1948). In the 1970s, Williams and colleagues developed further assessment tools to evaluate patients who were referred to long term care. They had observed that the undocumented and unsupervised referral of older patients to care homes often resulted in an unnecessary loss of autonomy which could be prevented by a structured assessment of the needs of the older person (Williams et al. 1973; Forciea 2008). From the beginning, the central aspect of geriatrics was the understanding that a comprehensive interdisciplinary assessment model is needed to see the patient as a whole, so that they receive adequate care (Forciea 2008).

Nevertheless, the discussion of the intertwining of the social and biological aspects of old age in medicine are still very much at the rudimentary stages (e.g. Calasanti 2005). Often old age is equated with illness and physical decline. Yet, the biological or genetic elements are only one aspect. As gerontological studies have shown, psychological and socio-cultural aspects likewise contribute to the experience of aging and old age (Anders 2009). Furthermore, it would be a mistake to think of older people as a homogenous group. Their physical and mental health status can range from being healthy and independent to being in need of constant care (Anders 2009). However, external factors, such as their socio-economic situation, marital status, social environment, and gender can influence how people age and how aging affects their health (Arber and Ginn 1993; Arber and Cooper 1999).

The CGA was designed in the 1980s and further developed over the last few decades to overcome the disease-orientated model of health care assessment and create an assessment which suitably fits the medical needs of the older patients (Palmer and Onder 2018). It is one of the cornerstones of modern geriatric care and constitutes a unique interaction of medical and social professionals, addressing the physical, psychological and social aspects of the health of older patients (Pilotto et al. 2017; Ellis et al. 2011). The geriatric patient is defined by geriatric-typical multimorbidity, being 70 or older (or in some cases 80 years or older) and affected by age-related vulnerabilities that might lead to further health issues, including the danger of chronical illness and an increased risk of losing their autonomy through their ailing health (Bundesarbeitsgemeinschaft der Klinisch-Geriatrischen Einrichtungen e. V. 2007). However, since the assessment is a complex, time-consuming, intense and expensive resource, the patients must be chosen with care, so that they benefit from the lengthy process and are not overwhelmed (Soriano 2007).

CGA requires a multidimensional and multidisciplinary diagnosis process and is encompasses a care plan and review schedule (Palmer and Onder 2018; von Renteln-Kruse 2009). There are five dimensions of the assessment: physical and mental health, functioning, social circumstances, and environment (Welsh et al. 2014). The assessment takes place as a three-step process; first, the appropriate patient is identified, second, the patient is screened and assessed, thirdly, the treatment plan is developed and implemented (Soriano 2007). The assessment aims to optimise the treatment and care, increase the patients’ independence and quality of life, and decrease unnecessary care and treatment (von Renteln-Kruse 2009; Ellis et al. 2011; Soriano 2007). CGA represents a patient-focused assessment instrument. The priorities are the patient’s needs, health, and quality of life which might contradict diseases-orientated models (Parker et al. 2018).

The assessment team is interdisciplinary in its composition and consists of medical doctors, nurses, physiotherapists, psychologists and social workers as well as occupational therapists, speech therapists, and if requested by the patient, spiritual guidance as well (Pilotto et al. 2017). However, variations in team composition and assessment dimensions can also be observed. The assessment can take place in an acute hospital setting, either in a geriatric unit or by a mobile assessment team, or in the outpatient setting by a GP (Ellis et al. 2011). Not every older patient needs a CGA. It is targeted at high-risk patients, but it is especially aimed at patients with complex patient history and with a view to improve their quality of life. However, although the complex health history of the patient is subject of CGA, other aspects of diversity that affect the health outcomes might still be overlooked as I show in the following. Furthermore, the reach of CGA is still limited. Although it is clear from research that many older people could benefit from an assessment through CGA, the use in practice is still somewhat limited due to the high costs as well as the expenditure of time of the assessment process (American Geriatrics Society 2006).

## Intersectionality and its implication for health care and medicine

In the late 1980s, Kimberlé Crenshaw developed the concept of intersectionality to understand multidimensional structural discrimination (Crenshaw 1989, 1990). Intersectionality describes the multicategorical intersection of social categories that determine structural discrimination that is not visible when only looking at one social category. Crenshaw based her theory on a case of workplace discrimination of Black women at General Motors. A group of Black women had sued General Motors for workplace discrimination, yet the judge ruled that no discrimination could be detected since General Motors employed (white) women and (male) Black people (Crenshaw 1989). Crenshaw argues that these Black women found themselves at the intersection of being Black and being women but were just being recognized by the law as one of both (Crenshaw 1989). Only through recognizing both social categories—race and gender—as non-hierarchical, equal intersecting identity markers was it possible to detect the structural discrimination these Black women faced in their workplace. Since then, intersectionality has sparked the interest of many feminist thinkers and developed into a concept that detects multicategorical, structural discrimination in relation to many social categories, such as class, sexuality, disability or age (Cho et al. 2013).

In many ways intersectionality is still a concept in the making. Intersectional perspectives come under many different labels, such as oppression, differences, multiculturalism, social inequalities and also diversity (Hearn and Louvrier 2015). However, diversity has been proven to be a challenging concept, since it lacks the binary component and only gains meaning with context (Hearn and Louvrier 2015). The result is that examining the impact of diversity can often be rather difficult to do in practice, as it is so broad. With intersectionality on the other hand, the focus is on the times, places and situations in which the intersections appear (Hearn and Louvrier 2015). This means that intersections are not fixed concepts but are very much related to the context. In this way, they provide the needed context for a diversity-sensitive approach to situations and the understanding of diversity through an intersectional lens. Since an intersectional approach pays particular attention to structures and how structures might lead to discrimination, it provides the needed context for the concept of diversity. In that sense, intersectionality focuses on a particular aspect of diversity that is not based on individual experience as such but relates individual experience to structures and structural discrimination. Intersectionality’s roots in the political movements of feminism and anti-racism make it a political tool to understand the effects of structures on diversity and how they create oppressions. Intersectional thinking places a strong emphasis on epistemic understandings of language and the effects of terms and structures, and how these might lead to invisible discrimination.

Medicine and health care have become important areas of intersectional research, because especially vulnerable groups often face undetected structural discrimination, for example with access to care and unjust treatment (e.g., Betancourt et al. 2003; Bowleg 2012; Hankivsky 2012; Beaudreau et al. 2019; Wilson et al. 2019). However, thus far the social category of aging and (old) age has received little attention in intersectional research in general but also in the medical and health context, so far. Older patients are still primarily considered as just old and not as a heterogenous group with diverse needs (Weßel and Schweda 2021). CGA as a standardized assessment tool provides the needed structures for the assessment of an older patient. Yet, these structures might (unintentionally) cause structural discrimination through the homogenization of patients, and render certain intersections of social categories invisible. Additionally, certain previous structures that caused discrimination and disadvantage for the patient in the part, might not be recognized during the assessment, resulting in a suboptimal outcome of the assessment for the patient. This would lead to structural discrimination through the assessment itself, which when made visible can be resolved by an intersectional approach.

## Identification and screening of the patient in the case of dementia

The cognitive assessment tools of CGA address whether the patient has a cognitive impairment that might affect their health and well-being or safety. However, the cognitive testing of older patients with complex backgrounds, especially in relation to class and race, may lead to false positive results (for example, see Gianattasio et al. 2019; Miles et al. 2001). Education and occupation are important social categories that influence cognitive impairment in old age as well as the outcome of cognitive tests that may lead to a diagnosis of cognitive impairment (Bloomberg et al. 2021; Miles et al. 2001). They are often intersected with racial aspects, which then create an even more complex intersection of social categories that need to be taken into consideration during the assessment and the interpretation of outcomes (Miles et al. 2001; Pan et al. 1999; Welsh et al. 1995).

Moreover, it is important to untangle this intersection carefully to understand the effects that the different social categories have, not just as added layers or factors in the analysis, but as actual, non-hierarchical intersections that contribute to the outcome of the CGA. Firstly, the intersection of education, race, class, and old age is an intersection which has cumulated over the patients’ lifetime (Dannefer 2003; Weßel and Schweda 2021). The intersection of race, education, and class and its effects on the person have been built up bit by bit, potentially starting with less formal education at a young age, resulting in an occupation that is often related to hard physical labour, and a low financial income (Garfield et al. 2008; Möller-Leimkühler 2003). A lower socio-economic status, stemming from a lack of education and occupation, impacts healthcare access and health literacy already at a younger age, and also influences the diagnosis and treatment of diseases in context of patient-doctor relationships (Wilson et al. 2019).

When comparing dementia rates of Black Americans and Caucasians in the United States, Black Americans have higher rates and more severe cases of vascular dementia, whereas Alzheimer’s dementia is more prevalent in Caucasian Americans (Miles et al. 2001; Gianattasio et al. 2019; Chin et al. 2011). Risk factors for vascular dementia such as hypertension, diabetes, and stroke are more prevalent in the Black community (Miles et al. 2001; Cheung et al. 2017). Cultural factors like the understanding of aging also play a role (Chin et al. 2011). Nevertheless, studies have shown that this racial difference is also related to a bias in cognitive test results due to an underestimation of educational and cultural factors (Chin et al. 2011; Welsh et al. 1995; Miles et al. 2001). The Black participants with fewer years of formal schooling were diagnosed with more severe dementia according to the test results (Welsh et al. 1995). This might be a twofold problem. On the one hand, higher education might have a preventive effect for dementia as some studies suggest (Livingston et al. 2020; Ngandu et al. 2007; Ott et al. 1995). On the other hand, there are also hints that the cognitive tests assume a universal level of education and that people with less formal education score worse, due to their education level and not because of severe dementia (Miles et al. 2001, p. 483).

Tests to assess the cognitive status of a person are standardized in a way that does not consider the life circumstances and various intersections that influence the patients’ health status. This lack of consideration on the part of the medical staff can influence the outcome of the CGA and potentially lead to the wrong diagnosis. If the patient is diagnosed with cognitive impairment or dementia, this might have an effect on the further development of the treatment plan and its implementation, for example in context of medication, care, and the assessment of the living situation. If indeed, as some studies indicate, factors like race, class, and education might influence the test, the further treatment of the patient might be negatively affected by the false positive results (Chin et al. 2011; Welsh et al. 1995; Miles et al. 2001). It is important to differentiate whether the reduced performance in a cognitive test is due to actual cognitive decline or because the patient did not have a standard level of education. A false positive result might lead to serious and irreversible consequences for the patient’s health due to misdiagnosis, inadequate treatment, or limitation of therapy.

## Development of a treatment plan with a frail patient

Frailty might be one of the leading factors that influences the treatment plan of the patient. The classification criteria of frailty are weight loss, slowness, weakness, exhaustion, and low physical activity (Szanton et al. 2009; Tatum et al. 2018). Frailty can be influenced by prior diseases, the patients’ physical status, and nutrition (Szanton et al. 2009). It is associated with a loss of independence in daily living, reduced physical activity, and a higher risk of falls and injury (Turner et al. 2015; Schoene et al. 2019; Körtner 2006). The testing for frailty is problematic in two ways, firstly social categories, such as the socio-economic status or gender, influence the health over the life course and in old age assessment of frailty. Secondly, the standardized testing overlooks the social impact on frailty and in this way provides incorrect assessments.

Yet, studies have shown that the assessment of a frailty status is influenced by several additional factors beyond the body’s biological health, such as gender, race, and class. Women are more often assessed as frail than men. Perhaps this is due to the fact that often women reach a more advanced age than men and frailty is related to advanced age and physical decline (Xue 2011). However, when only looking at the category of gender, the picture of frailty is too one-dimensional. When including more social categories like race, this ratio starts to change and reveals a more nuanced picture. Members of minorities find themselves more often in precarious socioeconomic living situations and their life is impacted by hard physical labour, a lack of health care access, poor nutrition or substance abuse due to the precarity of their life circumstances (Dannefer 2003; Bartley 2016). This results in a more frequent and earlier assessment as frail, disabled, and/or in need of admission to care, in comparison to people of the same age with a higher social status (Pan et al. 1999). The highest frailty rates in the United States can be found among Black women, followed by Black men, Hispanics, and Caucasian men and women (Hirsch et al. 2006; Szanton et al. 2010; Xue 2011). In addition to the socio-economic status, education seems to play a role in the assessment of frailty. Women without a high school degree are three times more likely to be diagnosed with frailty than women with a high school degree (Szanton et al. 2010). Women with less than $10,000 income per year are at twice the risk to be classed as frail in old age than women with higher incomes (Szanton et al. 2010).

The complexity of the status of frailty causes several concerns in the context of structural discrimination and intersectionality. The assessment of frailty is based on standardized testing (Rockwood 2005). The standardization of the testing raises the question how the assessment norms are established and how far it is possible to assess people outside the norm adequately. For example, Rockwood argues that frailty is considered as a wasting disorder that is related to weakness, slowness, low physical activity, exhaustion, and weight loss (Rockwood 2005, p. 1069). However, the focus on weight loss (also criticised by Reid et al. 2018) obscures the fact that obese older people might suffer from frailty as well, but might not be diagnosed as frail because of the assessment criteria (Rockwood 2005, p. 1069). The focus of certain standardized criteria might also be a problem in the context of other social categories, such as gender, race or education. The differences which occur through the intersections of various social categories might not be visible through standardized testing. This can then lead to a form of structural discrimination of people who are outside the testing standards, either as an over- or underdiagnosis.

Similar effects can also be observed in the medicalization of age-associated loss of abilities which are often considered as a form of frailty (Reid et al. 2018). Some older people who are assessed as frail consider this as failure or experience stigmatization due to the status (Reid et al. 2018; Gallagher and Cox 2019). First of all, many older people do not identify with the term frail on an individual level and this may impact their reaction to and in dealing with the status (Reid et al. 2018). This lack of identification however might not only be based on an individual understanding of aging but can also be rooted in the cultural or social understanding of aging and being old in the patient’s (and also the doctor’s) community. If, for example, the physical decline is understood as a natural part of aging in a cultural group, the status of frailty might not be considered as anything that requires medical care. Or if, for example, in context of gender, it is stigmatizing for older men to lose their physical strength, the status of frailty might lead to stigmatization in their group or to personal issues in dealing with their status.

From the beginning, frailty has been a socially influenced status that has become medicalized (Reid et al. 2018). The standardized assessment focuses on medical aspects, and the social elements do not receive as much attention as needed. Therefore, what is needed is an intersectional approach to the frailty assessment that considers the intersection of social categories as well as the structural effects of the assessment. This would help to create a more nuanced standardized CGA that pays attention to the possible experience of vulnerability, stigmatization and feelings of failure of the person assessed. A more nuanced assessment of frailty will have a significant influence on the development of the treatment plan. The incorrect assessment of a person as frail might lead to inadequate measures in the plan, for example in context of care or the housing arrangement. On the other hand, if the patient does not accept their frailty status, this can lead to the problem that the treatment plan is not accepted and implemented as it should be.

## Implementation of the care plan with non-heteronormative patients

The third part of CGA is the implementation of the treatment plan. This is a crucial aspect of CGA, since the implementation of a well-fitted treatment plan will, in the long-term, ensure the positive outcome of the CGA-process for the patient. The implementation of the treatment plan not only consists of the right medication but touches upon the patient’s lifestyle and their living environment. For example, if the patient previously had been assessed as cognitively impaired and as frail, it is crucial to know during the implementation of the plan whether the patient lives alone with no assistance, or in a robust social network in form of a family or a partner who might be able to provide care. To gain this information during the first stage of CGA, the social aspects are assessed, which includes gathering information about the social networks of the patients, for example, their marital status and family situation. Financial resources are likewise an important aspect of the implementation of the treatment plan. What financial resources exist to hire help, acquire assistive technology or fund the transfer to a care home, and how can this be financed if needed? Furthermore, the environmental adequacy and safety is considered; does the current environment the patient lives in suit their needs and can their safety be guaranteed (Pilotto et al. 2017; von Renteln-Kruse 2009).

In the context of the implementation of the plan, pitfalls often occur as older patients tend to be treated as a homogenous group. Additionally, it is important to assess the personal wishes and moral considerations of the patient (von Renteln-Kruse 2009), which can only be achieved if their identity and life circumstances are fully understood (Lottmann 2020; Misoch 2017). Heteronormativity, cultural or class differences can lead to a different understanding of personal wishes and moral considerations. The patient must be able to address these and the assessing party needs to be able to take them into consideration.

The assumed heterosexual identity of older people is one example of an issue that might occur in the implementation of the treatment plan. For example, when implementing the treatment plan, an assumed heterosexuality might lead to false assumptions about the support system of the patient (Lottmann 2020; Heusinger and Dummert 2016; Dune et al. 2020). Today’s older non-heterosexual generation grew up in a time when their non-heteronormativity was met with prejudice, medicalization, and criminalization, which has influenced the way they disclose their sexual orientation and also the way in which they organise their social lives (Tolley and Ranzijn 2006; Löf and Olaison 2020; Mahieu et al. 2019). Yet, regarding the aging LGBT community, studies have shown that in gerontology and care, heteronormative perceptions of older people are predominant and influence the way older people and their living environment are assumed to be (Dune et al. 2020; Harrison 2006; Tolley and Ranzijn 2006; Phillips and Marks 2006). Additionally, it has been observed that on the topic of sexuality and sexual orientation, a ‘don’t ask, don’t tell’-policy is still common (Dune et al. 2020; Phillips and Marks 2006).

However, they might have additional social networks outside the heteronormative spectrum, like an extensive circle of friends, who have taken on the role of family. During the implementation of the treatment plan, the non-heteronormative social structures and their value for the person must be known and understood to avoid possible discrimination. Wardecker and Johnston (2018) have developed a LGBT-friendly question catalogue for this purpose. The idea is to ask questions about the family and social background in a neutral, non-heteronormative way. For example, the assessor should not ask if the person will bring their husband/wife with them (for the assessment or any other appointment) but rather ask openly whom they have or will bring with them. Other forms of open and neutral questions are to ask if they have someone to take care of them at home instead of asking whether their family will take care of them, or to ask if they have a support system instead of asking if their children will support them (Wardecker and Johnston 2018). In this way, the questions are open to any answer and do not presume a particular lifestyle. The patient can feel comfortable to disclose these intimate parts of their life story, which are often connected to feelings of shame or previous experience of discrimination, so that the implementation of the treatment plan fits their needs and results in positive outcomes (Lottmann 2020; Dune et al. 2020). In this way, possible pitfalls based on heteronormative structures are paid attention to and structural discrimination based on these assumptions are avoided.

The treatment plan might result in two options regarding the living environment. The patient might either receive care at home or move into a care home because care at home is not a possibility due a lack of support or the complexity of their health issues. As discussed in the previous section, people from the margins are more frequently admitted to care homes than people with normative living environments. However, admission to a care home might not always be the best personal outcome, even if it is the best medical decision. Studies show that LGBT-people do not always feel comfortable in heteronormative-arranged care homes due to fears of stigmatization or discrimination (Leyerzapf et al. 2018; Lottmann 2020). Qualitative studies indicate that most of the older LGBT-people wish to be open and visible about their sexual orientation when they move into a care home (Löf and Olaison 2020). Some would prefer a LGBT-care home that is specialised to their needs instead of a heteronormative care home (Löf and Olaison 2020). Others wish to move into a common care home and expect that the staff educate themselves and that they are met without prejudice by the other residents (Löf and Olaison 2020).

## An intersectional approach to CGA and geriatric medicine

Gerontology, in particular feminist gerontology, has studied the intertwining of gender and age and concluded that age and gender are a strongly connected organisational principles that shapes the social and political, and also the biological and medical, circumstances of aging (Calasanti et al. 2006). Nevertheless, gender alone is a too limited perspective when discussing the diversity of older patients in geriatrics. Intersectionality suggests that identities cannot be understood one-dimensionally but must be seen as multi-dimensional and intersecting to understand structural discrimination. Despite the aim of intersectional approaches in medicine and health care research to improve the treatment of diversity and reduce structural discrimination, the results are somewhat limited. Thus far, the core categories of intersectionality—gender, race, and class—are still primarily the only social categories that are investigated (Bauer and Scheim 2019; Wilson et al. 2019; Bowleg 2012). However, in recent years sexuality and sexual orientation is becoming of increasing interest in intersectional approaches to health care and medicine (Beaudreau et al. 2019; Sangaramoorthy et al. 2017). Nevertheless, older people as such who face structural discrimination and inequality in medicine and healthcare, as well as also in other social contexts, have attracted little attention of intersectionality (Weßel and Schweda 2021). Despite the fact that geriatrics revolves around the biological and social categories of old age, the intersection with other social categories such as gender, race, or class, has received little attention until now. An intersectional approach can help to recognize the diverse identities and needs of the patient, as well as help to prevent discrimination and release patients from potential feelings of shame and hiding.

The standardized testing of the CGA primarily contributes to the problem of structural discrimination and an intersectional approach might be helpful to create a more sensitive and nuanced standardization. I have shown in the systematic discussion of the three assessment areas that the standardized testing for frailty, as well as the cognitive impairment and standardized questions in the social assessment do not grasp the diversity of the patient and might lead to a false diagnosis and ill-adjusted treatment plan. However, also an intersectionality-informed approach to CGA is a standardized tool. Yet, when using intersectionality, the focus in not so much to fit an individual into a norm but to address the structural discriminations which might arise from using a standardized tool. So, the goal is not to erase the standardization, but to address structural issues within their use. Thus, with the help of an intersectional approach the already existing tools of CGA can be improved to become more suitable for people who experience structural discrimination through the standardization.

Intersectionality can be a tool to create awareness of the complexity and partly hidden experience of people. The special focus on structural discrimination is particularly crucial in the use of intersectionality because it helps to shift the focus from the individual to structural dimensions that might lead to the discrimination of certain groups. In this way, the individual needs can be respected and also the structures that cause these needs to be ignored even devalued can be changed. Intersectionality helps to train the doctor or social worker to treat these parts of the assessment with special sensitivity in order to receive honest and open answers. False answers due to feelings of shame and experience of discrimination could jeopardise the assessment and its outcomes, and as such the assessment would fail to improve the quality of life of the patient. As a research method, intersectionality enables people define themselves and share their stories, rather than imposing identity categories on them (Bowleg 2008). This method of creating an open and trusting relationship between the assessor and patient can be helpful in allowing the patient to share the needed information.

Standardized testing can lead to discrimination because a standardization assumes a certain norm, which is based on the majority and often does not take into account minority perspectives or experiences. As such, when these particular life circumstances that influence a person’s health are unknown or not asked about, this can negatively skew the assessment’s outcomes. Additionally, with the current form of standardized testing and the issue of sensitive subjects, the focus on the medical aspects of the assessment can lead to equality issues. The dominance of medical professionals in the healthcare sector is also visible in multidisciplinary teams. Other healthcare professionals, such as care workers or social workers, are aware of this dominance and often state that it affects their autonomy and shows non-medical perspectives in a negative light (Gair and Hartery 2001). Geriatric care is defined by its multidisciplinary teams, and CGA is an example in which a multidisciplinary team must be able to contribute their perspectives equally, to ensure a truly inclusive and comprehensive assessment of the patient’s situation. However, the dominance of the medical perspective can be seen in many studies about CGA that mainly focus on the medical parts of the assessment and rarely critically review the social aspects (e.g. Ellis et al. 2011; Tatum et al. 2018; Soriano 2007).

In this case, an intersectional perspective would not only strengthen the assessment of the patients but also improve CGA itself. In a way, CGA is in and of itself an intersectional assessment tool in which three healthcare aspects, the physical, the psychological, and the social intersect and interlock, rather than being hierarchical or additive. To take an intersectional perspective with the three elements of CGA would help to ensure that they are all treated equally during the assessment process and any dominance of the medical perspective would be avoided. The equality within the team is an important pre-condition to ensure an equal evaluation process that respects the importance of all three elements of the assessment as valuable, in order to create a comprehensive assessment of the patient and a diversity-sensitive treatment plan.

## Conclusion

Geriatrics is a discipline which aims to take a holistic approach to the aging body, and go beyond the examination and treatment of the physical and biological components of aging. In the context of the increasing knowledge of the diversity of older people, it is important to create awareness of the multiple experiences that older people have and how these can influence the treatment and care of older patients. This is significant because an intersectionally-informed treatment of older patients prevents structural discrimination and contributes to an adequate and just treatment of one of the most vulnerable groups in health care—older people with diverse identities.

CGA has been proven to be a tool which is inclusive in the way that it connects physical, psychological, and social aspects of the health of older patients, and its effect on the quality of life and health outcomes. In that way, it contributes to the diversification of perspectives on older patients and their health care needs. However, despite assessing age, gender, physical and mental health as well as the social environment, the standardized testing methods constitutes a challenge. They do not help to raise awareness of the diverse backgrounds of the assessed patients, but instead can create new inequalities, which might lead to unclear results, false diagnoses and a treatment plan that is not beneficial for the patient. These new inequalities can lead to further injustice and discrimination of older people in healthcare. Diversity might be acknowledged but not in a sufficient way that contributes to just healthcare.

Additionally, the assessment team must be trained to recognize the markers of intersecting identities and the effects of these intersections on the health of the patient. On the one hand, they must understand how different social categories intersect and how that might influence the assessment process. On the other hand, the team must be aware of the difficulties people with intersecting marginalised identities face during the assessment, such as understanding the test procedures or the implementation of the treatment plan. This requires special diversity training for the CGA team. This should not only be addressed to the professionals who conduct the assessment but to all team members, since intersecting identities and structural discrimination play a role in all three steps of the CGA process.

However, challenges occur in the introduction of intersectional methods into CGA. Firstly, CGA is a clinical tool and focuses on the medical aspects of health, whereas intersectionality is a sociological theory and methodology. Although intersectionality has been used in the context of public health (Hankivsky and Christofferson 2008), there is little known yet as to how it would work in a patient-centred clinical setting (Weßel and Schweda 2021; Wilson et al. 2019). Here several adjustments in the intersectional methods might be required to fit the clinical needs. However, intersectionality itself is not methodologically clear. For example, the selection of relevant categories still constitutes a challenge for any intersectional analysis as the long-standing neglect of ageing and old age shows.

Nevertheless, intersectionality supports an understanding and awareness of the pitfalls of the standardized tools in CGA and how they can disadvantage patients with diverse identities, even when the aim is an individual patient-centred approach to medical treatment. It contributes to ethical debates about injustice and discrimination in healthcare by drawing attention to the especially vulnerable group of older people, and the lack of recognition of their intersectional diversity that contributes to a multidimensional marginalization of their needs in geriatric medicine.

I suggest that intersectionality is a tool that can raise awareness of structural discrimination and intersecting identities in geriatrics generally, and during the CGA process specifically. Intersectionality can serve here as normative matrix to detect multidimensional marginalization of older people and health inequality in geriatric medicine. Additionally, it can serve as a guide on how to interact with people from the margins and help train professionals not to make assumptions regarding their patients’ identity, but instead give them the confidence to share their story themselves.

## Data Availability

Not applicable.
